# Phosphaturic Mesenchymal Tumor of the Greater Trochanter: A Case Report

**DOI:** 10.7759/cureus.70721

**Published:** 2024-10-02

**Authors:** José Pablo Bibiloni Lugo, Hector A Muñoz-Miró, Rafael Fernandez-Soltero, Norman Ramírez-Lluch, Juan Bibiloni

**Affiliations:** 1 Orthopedic Surgery, Ponce Health Sciences University, Ponce, PRI; 2 General Surgery, University of Puerto Rico, Medical Sciences Campus, San Juan, PRI; 3 Pediatric Orthopedic Surgery, Mayagüez Medical Center, Mayagüez, PRI; 4 Orthopedic Surgery, University of Puerto Rico, Medical Sciences Campus, San Juan, PRI

**Keywords:** hypophosphatemia, phosphaturia, phosphaturic mesenchymal tumor, s100 protein, tumor induced osteomalacia

## Abstract

This is the case of a 56-year-old Hispanic male with a history of multiple fractures and electrolyte abnormalities, including hypophosphatemia and phosphaturia. Physical examination, imaging studies, and laboratory workup may have suggested the presence of a phosphaturic mesenchymal tumor (PMT) causing osteomalacia. The patient underwent surgery for en bloc tumor removal, and the histopathological analysis confirmed the presence of neoplastic cells consistent with PMT with minimal immunohistochemical positivity to S100 protein, which is atypical for this type of tumor.

This case highlights the challenges in diagnosing PMTs due to their rarity and variable presentation. It emphasizes the importance of considering PMT in the differential diagnosis for unexplained hypophosphatemia and osteomalacia-like symptoms, especially in persistent disease after parathyroidectomy for presumed primary hyperparathyroidism. The atypical immunohistochemical profile observed in this case contributes to the growing body of knowledge about the heterogeneity of PMTs and underscores the need for comprehensive diagnostic approaches in suspected cases.

## Introduction

Phosphaturic mesenchymal tumor (PMT) is a rare neoplasm associated with tumor-induced osteomalacia (TIO), characterized by renal phosphate wasting and hypophosphatemia [1]. This case report presents a 56-year-old male with a history of multiple fractures, muscle weakness, persistent hypophosphatemia, and elevated urinary phosphate with osteomalacia that persisted despite parathyroidectomy performed for a presumed diagnosis of primary hypoparathyroidism. Upon presentation, the physical exam revealed muscle weakness and significant bilateral knee and ankle pain. Imaging studies revealed multiple pathologic fractures compatible with osteomalacia. A positron emission tomography (PET/CT) and magnetic resonance imaging (MRI) demonstrated a right greater trochanter lesion. Due to the laboratory, imaging, and persistent clinical findings, as well as the small size of the lesion, the patient underwent an en bloc excisional biopsy without complications. The histopathological examination revealed a richly vascular spindle cell neoplasm with bland cytology, consistent with a PMT associated with oncogenic osteomalacia [1]. Immunohistochemical studies showed the neoplastic cells were minimally positive for S100 protein, which is atypical for PMTs [2,3]. This case highlights the challenges in diagnosing PMTs due to their rarity and variable presentation. It also emphasizes the importance of considering PMT in the differential diagnosis for unexplained hypophosphatemia and osteomalacia-like symptoms. The atypical immunohistochemical profile observed in this case contributes to the growing body of knowledge about the heterogeneity of PMTs and underscores the need for comprehensive diagnostic approaches in suspected cases.

## Case presentation

**Figure 1:**
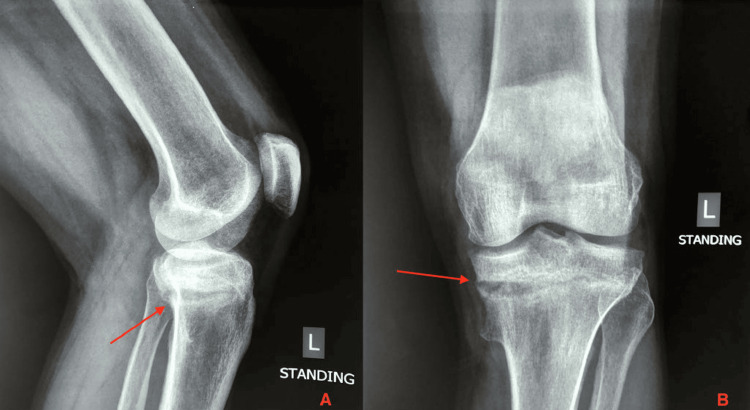
Left knee lateral (A) and AP (B) X-ray. Proximal tibia fracture was also present upon presentation.

**Figure 2:**
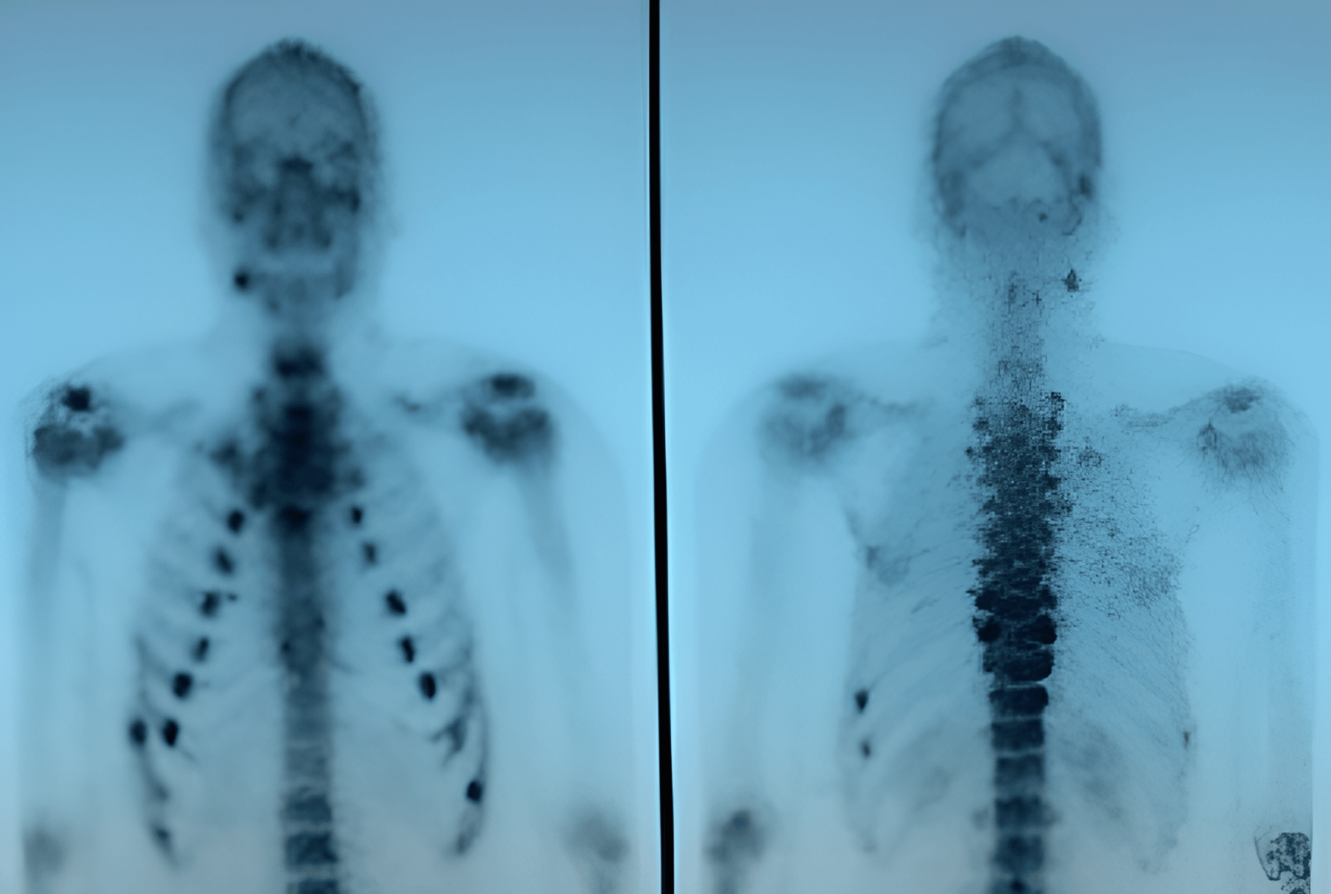
Three-phase technetium bone scan. Demonstrated costal rachitic rosary sign, a typical finding in patients with osteomalacia.

A 56-year-old male of Puerto Rican descent with a history of primary hyperparathyroidism status post parathyroidectomy, benign prostatic hyperplasia, and hypertension presented with multiple insufficiency fractures (Figures 1-2). A three-phase technetium bone scan showed results consistent with osteomalacia. The patient's family history was remarkable. On physical examination, his height was 1.93 m, weight was 110.7 kg, and body mass index was 27.9 kg/m^2^. His blood pressure was 150/80 mmHg. On initial presentation, the physical examination revealed bilateral knee and ankle pain, more pronounced on the right side, along with generalized muscle weakness. These symptoms were severe, making the patient wheelchair-bound.

**Figure 3:**
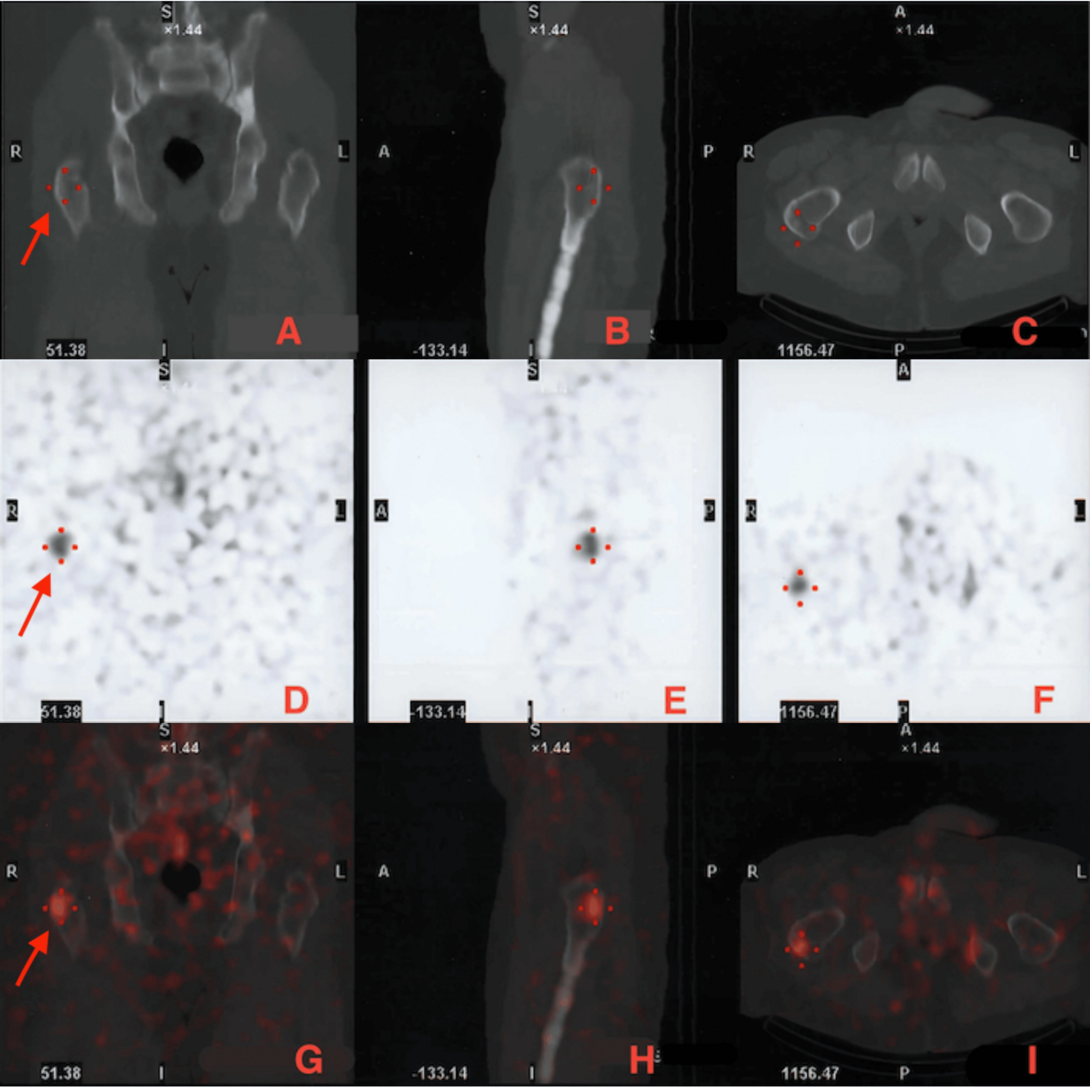
Whole body SPECT/CT. Coronal, sagittal, and transaxial CT (A, B, C) demonstrated an ill-defined irregular area of sclerosis in the intramedullary aspect of the right femur, in the region of the greater trochanter. Coronal, sagittal, and transaxial SPECT CT (D, E, F) show an area of increased uptake in the right femur greater trochanter. The fused SPECT/CT image (G, H, I) accurately establishes a correlation between the area of focal sclerosis observed on CT with the area of increased uptake observed on SPECT. SPECT: single photon emission computed tomography

**Figure 4:**
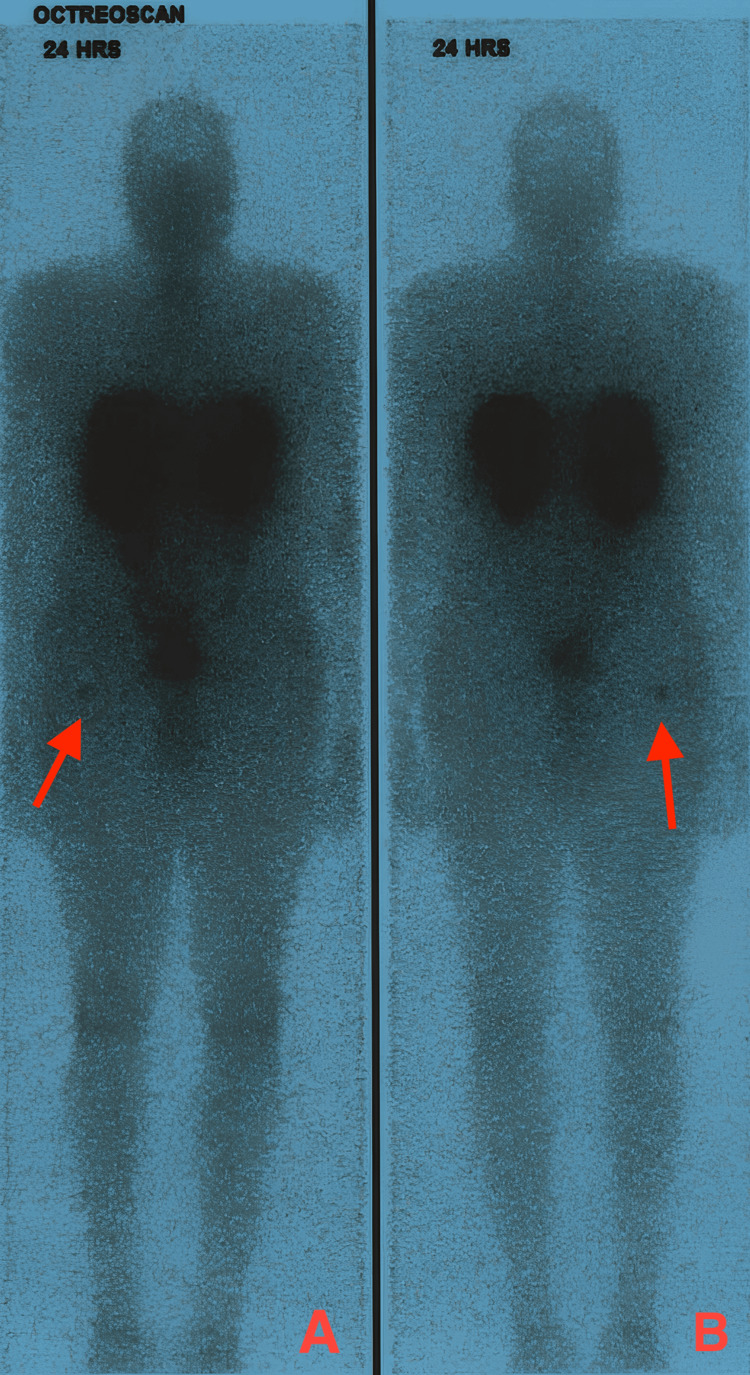
Whole body octreoscan anterior (A) and posterior (B) view. Small somatostatin receptor-rich lesion in the intramedullary aspect of the right femoral greater trochanter.

Laboratory results while patient on medical treatment for hyperparathyroidism showed upper range normal calcium level to low hypercalcemia at 10.58 mg/dL (normal range: 8.60-10.20 mg/dL), low phosphate at 2.38 mg/dL (normal range: 2.70-4.50 mg/dL), parathyroid hormone (PTH) at 63.5 pg/mL (normal range: 15-65 pg/mL), alkaline phosphatase (ALP) at 102 U/L (normal range: 40-128 U/L), and high levels of urine phosphate output of 1764 mg/24hr (normal range: 390-1425 mg/24hr). A right hip X-ray was unremarkable. However, a three-phase technetium bone scan demonstrated a costal rachitic rosary sign, a typical finding in patients with osteomalacia (Figure 2) [4]. A whole-body octreoscan and single photon emission computed tomography/computed tomography (SPECT/CT) scan were ordered, which revealed minimal uptake in the right femur greater trochanter (Figures 3-4).

The patient was evaluated for tumor-induced osteomalacia (TIO), including tests for serum phosphorus at 2.38 mg/dL (normal range: 2.70-4.50 mg/dL), urine phosphate at 1764 mg/24hr (normal range: 390-1425 mg/24hr), serum 1,25-dihydroxy vitamin D at 51.7 nmol/L (normal range: 18-64 pg/mL), and serum FGF23 levels at 1600 pg/mL (normal range: 19.9-52.9 pg/mL), all consistent low phosphate levels (see Table 1).

**Table 1:** Pertinent laboratory findings upon presentation.

Parameters	Patient values	Reference range
Serum phosphorus	2.38 mg/dL	2.70-4.50 mg/dL
Urine phosphate	1764 mg/24hr	390-1425 mg/24hr
Serum 1,25-dihydroxy vitamin D	51.7 nmol/L	18-64 pg/mL
FGF23	1600 pg/mL	19.9-52.9 pg/mL

**Figure 5:**
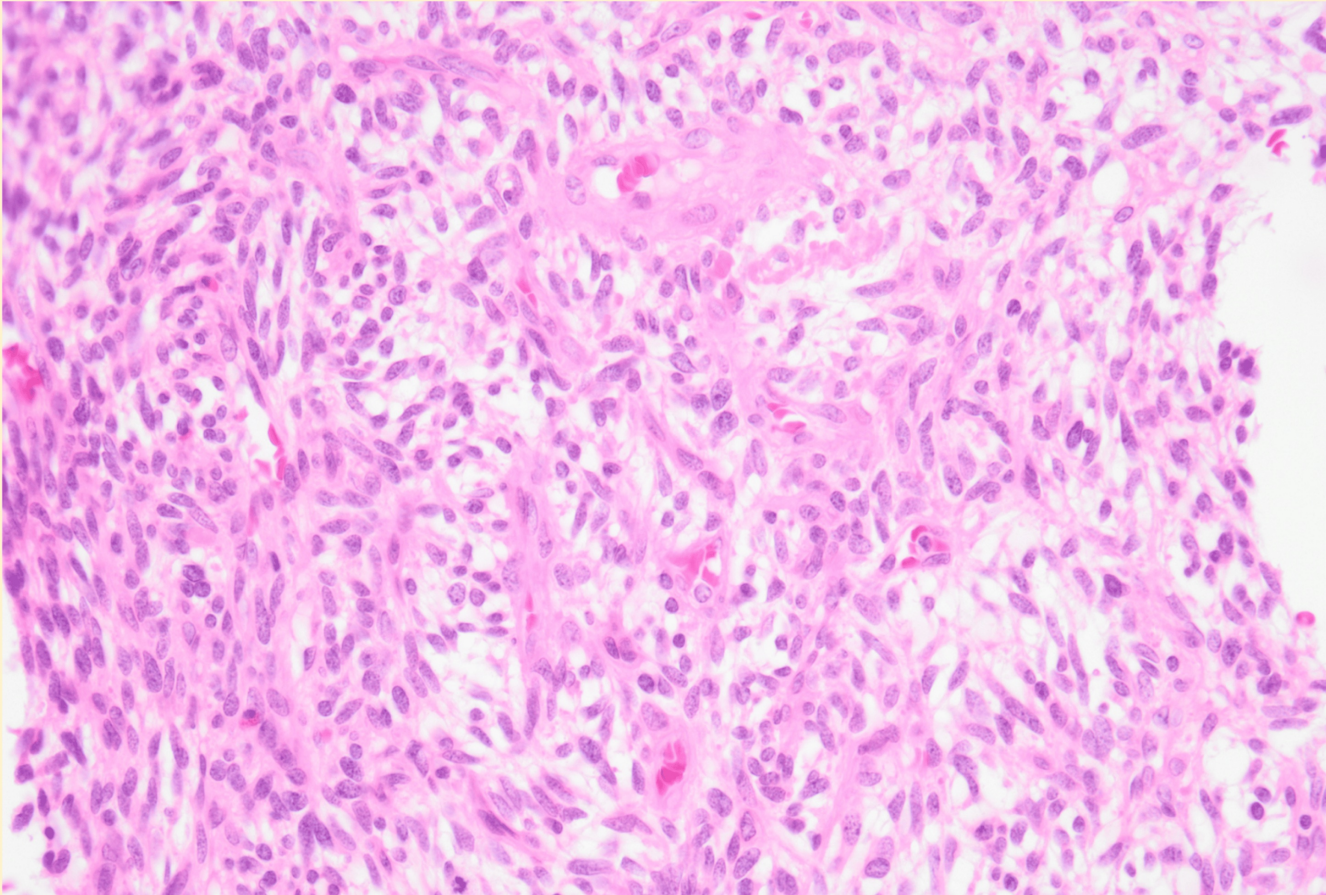
Histological analysis of this tumor revealed a richly vascular spindle cell neoplasm with bland cytology and rich vasculature.

The patient was scheduled for an en-bloc excisional biopsy of the right trochanteric lesion due to the small size of the lesion and suspicion of TIO. Histological analysis revealed a segment of his right greater trochanter measuring 2.6 x 2.2 x 1.5 cm with a tan-brown, coarse, rubbery texture. Microscopically, the tumor measured 7 mm, and the histology revealed a richly vascular spindle cell neoplasm with bland cytology and rich vasculature without calcifications, consistent with a PMT [1] associated with oncogenic osteomalacia (Figure 5). Immunohistochemical studies performed at the National Institutes of Health (NIH) demonstrated neoplastic cells minimally positive for S100 protein and negative for CD34, desmin, and smooth muscle antibody (SMA). These findings were consistent with the typical histopathological appearance of PMTs characterized by rich vascularity [1], with the exception of low levels of S100 protein, which is atypical for this tumor [5].

During follow-up evaluations, the patient's laboratory results continued to show hypophosphatemia, normal to high calcemia, and the development of elevated PTH levels, despite the absence of new physical complaints. Currently, the persistent biochemical abnormalities are being managed conservatively with oral phosphate supplementation and calcitriol. Despite these ongoing issues, the patient reports only minimal joint pain, maintains independent ambulation, and successfully performs activities of daily living without assistance.

## Discussion

Hypophosphatemia is a medical condition characterized by low levels of phosphate in the blood. Phosphate is an essential mineral that plays a crucial role in various physiological processes, including bone formation, energy metabolism, and acid-base balance. There are three primary mechanisms that result in hypophosphatemia: decreased intestinal absorption, cellular shifting of phosphate from the extracellular to intracellular space, and increased renal excretion [6]. Symptoms of hypophosphatemia are nonspecific but may include weakness, fatigue, muscle pain, and, in severe cases, respiratory failure, heart failure, bone fractures, and long bone bowing [6].

TIO is a rare paraneoplastic syndrome characterized by excessive phosphate excretion. TIO can be caused by mesenchymal tumors such as HPC, osteosarcoma, giant cell tumors, and other PMTs [7]. In the case of PMT, the resulting hypophosphatemia is due to high levels of fibroblast growth factor-23 (FGF23), a hormone that blocks the synthesis of 1,25-dihydroxycholecalciferol by inhibiting 1-α-hydroxylase and increasing renal phosphate excretion [7]. This hypophosphatemia leads to bone resorption to compensate for the electrolyte imbalance, eventually inducing an osteomalacia state in the patient.

PMT is a rare neoplasm associated with TIO, with approximately 450 cases reported globally, including 12 Hispanic patients in the last decade [1]. PMT is characterized by renal phosphate wasting, hypophosphatemia, and osteomalacia (softening of the bones) [8]. This type of mesenchymal tumor typically produces FGF23, a hormone crucial for phosphate homeostasis, leading to musculoskeletal deficiencies [1]. This disturbance in mineral metabolism causes TIO, with symptoms as described above [8]. On average, clinical symptoms appear at least three years before diagnosis and persist for over five years after tumor resection [1].

Diagnosing PMTs requires a comprehensive approach combining clinical, biochemical, imaging, and histological methods [2]. As described previously, patients typically present with symptoms of TIO. Biochemical tests reveal characteristic findings including hypophosphatemia, elevated serum FGF23 levels, low or inappropriately normal 1,25-dihydroxyvitamin D levels, and elevated alkaline phosphatase [3,9]. Findings that are consistent with this diagnosis (Table 2) [9].

**Table 2:** Biochemistry in phosphaturic mesenchymal tumors with concomitant TIO. Copyright/license: This table has been adapted from an open-source article [9] distributed under the terms and conditions of the Creative Common CC BY license. TIO: tumor-induced osteomalacia; FGF23: fibroblast growth factor-23

Laboratory parameter	Expected alteration
Serum phosphate	Decreased
Urinary phosphate	Inappropriately normal or increased in relation to low serum phosphate
Serum calcium	In the lower half on the normal range or slightly decreased
Serum alkaline phosphatase (ALP)	Increased
FGF23 (intact)	Increased or inappropriately normal
FGF23 (C-terminal)	Increased
1,25(OH) vitamin D	Decreased or inappropriately normal in relation to low serum phosphate
25(OH) vitamin D	Variable. It depends on dietary intake and sun exposure

Imaging is essential for PMT diagnosis, utilizing various modalities. Radiologically, PMTs often exhibit indistinct margins and may resemble benign bone lesions or osteomalacic changes. Furthermore, PMTs can occur at various anatomical sites, further complicating their recognition solely based on clinical and radiological grounds. Contrast-enhanced computed tomography (CT) scans often highlight fat or calcification in the tumor. MRI shows PMTs as isointense on T1-weighted and hyperintense on T2-weighted sequences. For comprehensive whole-body assessment, gallium-68 DOTATATE PET is the preferred nuclear imaging technique [1].

Identifying the tumor and confirming its association with TIO can be challenging due to the small size and often inconspicuous nature of PMTs. Surgical removal of the tumor is the primary treatment for PMT, and successful resection typically leads to the resolution of TIO symptoms, although this may take up to five years [1]. Locating the tumor often requires thorough preoperative localization using various imaging modalities to ensure better surgical outcomes [2]. Achieving negative margins after excision is crucial for better outcomes and symptomatic resolution, as well as for reducing the risk of recurrence [2]. Serum FGF23 levels can serve as a useful diagnostic tool and a means to monitor for tumor recurrence [1].

In histopathology, PMTs have hemangiopericytoma (HPC)-like vasculature with minimal mitotic activity [1]. They are composed of various cell types, including spindle cells, osteoclast-like giant cells, and occasionally chondrocytes or adipocytes. Additionally, grungy calcifications and microcystic changes may often be noted. Immunohistochemically, PMTs often show activity for FGF23 and, in some cases, somatostatin receptor 2A (SSTR2A) [5]. Neither of these findings is specific for establishing a diagnosis of PMT, as these can be positive in aneurysmal bone cysts, osteosarcomas, synovial sarcomas, and hemangiomas, as well. FGF23 expression can be detected by reverse-transcription polymerase chain reaction, chromogenic in situ hybridization, or immunohistochemistry [10]. Notably, this case presents an atypical immunohistochemical profile, with the tumor cells showing minimal positivity for S100 protein. This finding contrasts with the typical immunohistochemical characteristics of PMTs, which usually lack neurosecretory markers such as S100 [2,5]. Several S100 members, mainly S100A4 and S100A8/9, have been identified as key players in the pathogenesis of many types of cancer, as well as of several other disease conditions, including diabetes and other inflammatory diseases. S100 proteins have multiple functions, which include regulation of proliferation, differentiation, apoptosis, Ca2+ homeostasis, energy metabolism, inflammation, and migration/invasion of tissues [11]. The presence of S100 in addition to this type of tumor should result in a low level of serum calcium. In this particular case, the patient presented with low-grade hypercalcemia. This could be a consequence of chronic calcitriol use. This variation emphasizes the heterogeneity of PMTs and the need for comprehensive histopathological examination in diagnosis.

When considering the histological differential diagnosis for this tumor, several entities come into play: solitary fibrous tumor, mesenchymal chondrosarcoma, and paraganglioma. Solitary fibrous tumors share some similarities, such as staghorn vessels and a benign spindle cell appearance. However, they can be distinguished from PMTs by the absence of characteristic matrix and calcifications, as well as the lack of SSTR2A or FGF23 mRNA expression. Mesenchymal chondrosarcomas differ in that they exhibit malignant, small, round cells. They also lack the distinctive calcified matrix typical of PMTs and do not express FGF23 mRNA. Paragangliomas, particularly those that are highly vascular, may occasionally resemble PMTs morphologically. However, the immunophenotype of PMTs (positive for SSTR2A and FGF23 mRNA, negative for chromogranin and synaptophysin) rules out this possibility. Additionally, while both tumors can be highly vascular, paragangliomas lack the characteristic chondromyxoid matrix and calcifications seen in PMTs. Paragangliomas also display a distinctive nested (zellballen) architecture comprised of neuroendocrine cells and show positive staining for neuroendocrine markers such as synaptophysin and chromogranin, features not observed in PMTs [12].

Rare instances of histologically malignant PMTs have been documented in medical literature. These unusual cases exhibit clear sarcomatous characteristics, including elevated nuclear grade, increased cellular density, areas of necrosis, and heightened mitotic activity. The appearance of these malignant PMTs often resembles adult-type fibrosarcoma or undifferentiated pleomorphic sarcoma. Interestingly, in most of these malignant cases, pathologists can typically identify a pre-existing component that appears benign. This benign-looking element may be found within the primary tumor itself or in tissue from an earlier presentation of the disease. This observation suggests a potential progression from benign to malignant forms in some PMTs. It's worth noting that unlike their benign counterparts, malignant PMTs do not seem to produce significant amounts of matrix. This absence of matrix production appears to be a distinguishing feature of the malignant variant, potentially aiding in its identification and differentiation from benign PMTs [1].

This case report highlights the challenges in diagnosing and treating PMTs, a rare neoplasm associated with TIO. The patient's persistent hypophosphatemia, phosphaturia, history of multiple fractures, and physical exam findings were key indicators that led to further investigation and eventual histologic diagnosis of PMT.

The case underscores the importance of considering PMT in the differential diagnosis for patients presenting with unexplained hypophosphatemia and osteomalacia-like symptoms [1]. The successful tumor localization, using a combination of SPECT/CT, MRI, and octreoscan, demonstrates the value of multimodal imaging in identifying these often-elusive tumors [8].

The successful surgical resection of the tumor aligns with the current standard of care for PMTs, where complete excision is considered curative [13]. Long-term follow-up, including monitoring of urinary phosphate levels and FGF 23, among other pertinent laboratories, as well as periodic imaging, will be crucial to ensure the absence of recurrence and complete resolution of symptoms.

## Conclusions

This case report highlights the diagnostic challenges and importance of identifying PMTs, a rare neoplasm associated with TIO. The successful tumor localization and surgical resection demonstrate the value of a comprehensive, multimodal approach utilizing clinical, biochemical, imaging, and histopathological methods. While PMTs typically exhibit characteristic features like rich vascularity and FGF23 positivity, the atypical S100 protein expression, in this case, underscores the heterogeneity within this disease entity. Maintaining a high index of suspicion for PMT in patients presenting with unexplained hypophosphatemia and osteomalacia-like symptoms, along with close long-term monitoring following curative tumor resection, is crucial for optimal management of this rare but clinically significant disorder.
